# Advances in Synthesis and Applications of Self-Healing Hydrogels

**DOI:** 10.3389/fbioe.2020.00654

**Published:** 2020-07-21

**Authors:** Leqi Fan, Xuemei Ge, Yebin Qian, Minyan Wei, Zirui Zhang, Wei-En Yuan, Yuanming Ouyang

**Affiliations:** ^1^Department of Orthopedics, Shanghai Jiao Tong University Affiliated Sixth People’s Hospital, Shanghai, China; ^2^Engineering Research Center of Cell & Therapeutic Antibody, Ministry of Education, and School of Pharmacy, Shanghai Jiao Tong University, Shanghai, China; ^3^Shanghai Sixth People’s Hospital East Affiliated to Shanghai University of Medicine & Health Sciences, Shanghai, China; ^4^School of Light Industry and Food Engineering, Nanjing Forestry University, Nanjing, China

**Keywords:** self-healing, hydrogel, repair mechanism, biomedical application, biomaterials

## Abstract

**Background:**

Hydrogels, a type of three-dimensional (3-D) crosslinked network of polymers containing a high water concentration, have been receiving increasing attention in recent years. Self-healing hydrogels, which can return to their original structure and function after physical damage, are especially attractive. Some self-healable hydrogels have several kinds of properties such as injectability, adhesiveness, and conductivity, which enable them to be used in the manufacturing of drug/cell delivery vehicles, glues, electronic devices, and so on.

**Main Body:**

This review will focus on the synthesis and applications of self-healing hydrogels. Their repair mechanisms and potential applications in pharmaceutical, biomedical, and other areas will be introduced.

**Conclusion:**

Self-healing hydrogels are used in various fields because of their ability to recover. The prospect of self-healing hydrogels is promising, and they may be further developed for various applications.

## Introduction

Hydrogels are a type of 3-D chemically or physically cross-linked polymeric network with a high water content. They have a wide range of uses in biomedical fields. However, as soft materials, many hydrogels tend to be damaged or fatigued easily. Self-healing hydrogels are hydrogels that are able to recover their structures and restore their functions after physical damage either in an autonomic way or by relying on external stimuli. Some self-healing hydrogels have additional properties, such as injectability and conductivity, which enable them to be further used in several areas, including cell/drug delivery, tissue engineering, soft electronic devices, and so on.

Self-healing mechanisms can be divided into intrinsic ones and extrinsic ones. The mechanism of intrinsic type self-healing hydrogels is mainly attributed to the reversible networks formed by the polymers themselves. Those networks can be mainly divided into two cross-linking types: (1) dynamic chemical covalent bonds and (2) physical non-covalent interactions. Dynamic covalent bonds include imine bond (Qu et al., 2017; Yan et al., 2017; Chen H. et al., 2018; Chen et al., 2019e; Khan et al., 2018; Wei and Gerecht, 2018; Zhang et al., 2018; Guo B. et al., 2019; Jiang F. et al., 2019; Li Q. et al., 2019; Qian et al., 2019; Wang et al., 2019b; Wang W. et al., 2019; Zhou et al., 2019), Diels–Alder (DA) reaction (Oehlenschlaeger et al., 2014), boronate-diol complexation (Hong et al., 2018; Pan et al., 2018; Smithmyer et al., 2018; Zhi et al., 2018; Han et al., 2019), acylhydrazone bond (Yang et al., 2017; Shen et al., 2019; Sun C. et al., 2019), dynamic disulfide bond (Song L. et al., 2019), and dynamic metal oordination (Shao et al., 2018; Chen et al., 2019a; Qin et al., 2019). Physical non-covalent interactions include hydrogen bonding (Fan et al., 2018; Uzumcu et al., 2018; Cao et al., 2019; Chen et al., 2019c; Hu et al., 2019; Huang et al., 2019; Kuddushi et al., 2019; Li R. et al., 2019; Liao et al., 2019; Su et al., 2019; Wang L. et al., 2019), hydrophobic interaction (Liu P. et al., 2019), host-guest interaction (Deng Z. et al., 2018; Li Q. et al., 2018; Liu L. et al., 2019; Wang W. et al., 2019), and ionic interaction (Ma et al., 2018; Wang Y. et al., 2018; Becher et al., 2019). Some intrinsic type self-healing hydrogels even have several kinds of networks, containing either one type or several types of mechanisms. In addition, some intrinsic type self-healing hydrogels consist of materials that can heal by themselves under certain circumstances (Lee et al., 2018), and thus achieve self-healing. There are also some self-healing hydrogels that do not rely on the reversible bonds or interactions of the polymers themselves to recover; instead, healing agents are stored in reservoirs and embedded in the polymer matrix to aid the self-healing (Chen et al., 2019d; Liu S. et al., 2019; Rao et al., 2019). These kind of hydrogels are called extrinsic type self-healing hydrogels.

In this review, some recent research in self-healing hydrogels will be explained, including the mechanism and synthesis of self-healing hydrogels. The emphasis of this review will be put on intrinsic type self-healing hydrogels, and the extrinsic type will also be briefly introduced. In addition, the application of these materials will be introduced, such as their use in cell/drug delivery, or as glues, sensors, and wound healing materials, and future potentials of self-healing hydrogels will be discussed.

## Mechanism of Intrinsic Type Self-Healing Hydrogels

**FIGURE 1:**
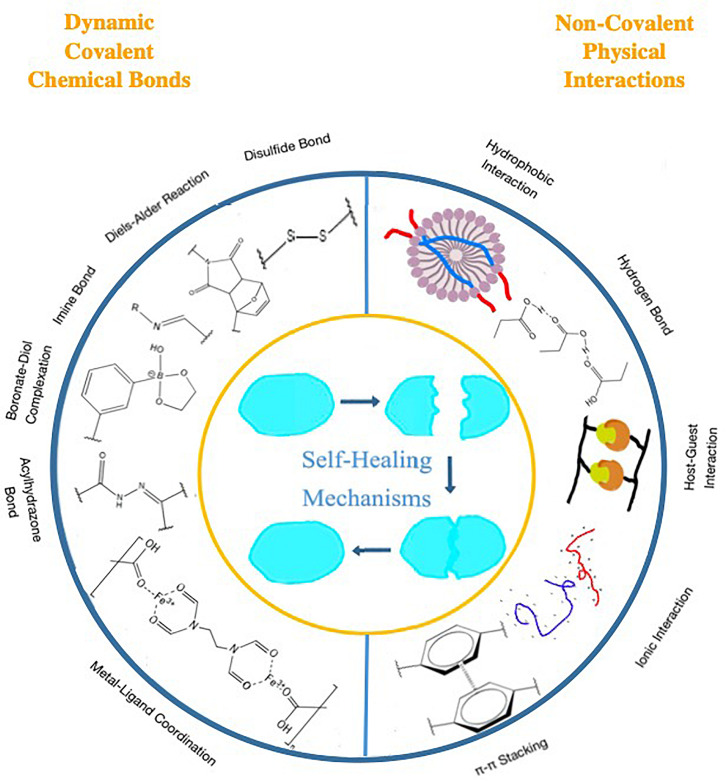
Major dynamic chemical covalent bonds and physical non-covalent interactions as self-healing mechanisms.

Most intrinsic self-healing hydrogels are synthesized by either one or both types of interactions: dynamic chemical covalent bonds and physical non-covalent actions. Figure 1 shows some major types of these two kinds of mechanisms.

There are also some exceptions of self-healing hydrogels. Hydrogels that consist of certain materials are able to recover by themselves in certain conditions. For example, self-healing can be achieved due to gelatin’s denaturation with temperature changes.

### Dynamic Chemical Covalent Bonds

#### Disulfide Bond

Being strong and sensitive to exchange reactions in various conditions, such as heating (Canadell et al., 2011), a disulfide bond was used in rubbers to obtain self-healing properties (Canadell et al., 2011; Xiang et al., 2019). The disulfide exchange can occur and repeat multiple times under several circumstances, including heating, photoirradiation, and mechanical stress. Song L. et al. (2019) used this kind of bonding to develop a multi-responsive, self-healing hydrogel with a single component. Pluronic F127, a thermo-responsive copolymer, and LA, an antioxidant, were first conjugated. The hydrogel was then formed by cross-linking the F127-LA polymer, starting with self-assembly into micelles, and was followed by disulfide exchange induced by ultraviolet (UV) light. The prepared hydrogel could respond to temperature and reduction, as well as realize self-healing. Stress-relaxation experiments and stress-strain curves of the F127-LA hydrogel showed that the mechanical properties, such as tensile strength, could be influenced by gel concentration or tensile rate; cytocompatibility assay showed that the hydrogel would not have a negative impact on cellular metabolism.

#### Diels–Alder (DA) Reaction

Diels–Alder covalent bonds can be dynamic under physiological conditions (Chen et al., 2002) and have thermal reversible characteristics (Oehlenschlaeger et al., 2014). DA kinetics is slow; as a result, it is easy to handle (Ghanian et al., 2018). This may be the reason why many researchers have combined DA covalent bonds with physical non-covalent interactions to prepare DN hydrogels. Ghanian et al. used furan substituted alginate and maleimide functionalized four-arm PEG cross-linker to trigger a DA click reaction (Ghanian et al., 2018). Calcium was also bound to the alginate to form an ionic interaction. The ionic interaction was sacrificed under loading, and the covalent DA bonds could thus avoid severe plastic deformation. Once the load was released, the DA bonds would guide the gel back to its initial position. Besides its self-healing ability, these two networks also made the hydrogel tough, moldable, and easily accessible for injection.

#### Imine Bond

**FIGURE 2:**
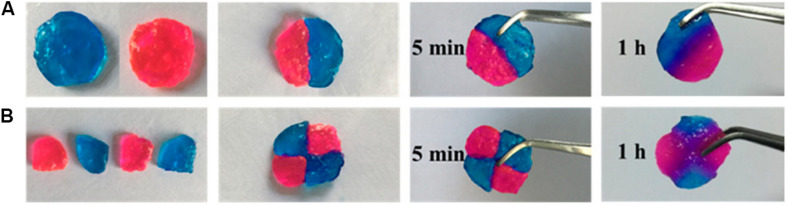
The process of self-healing of **(A)** Two pieces or **(B)** Four pieces of hydrogels prepared by Zhang et al. (2018) (reproduced with permission from Zhang et al. (2018). Copyright 2018 American Chemical Society).

Imine bond [sometimes also called Schiff base (Meyer et al., 2007)] is a reversible covalent bond prepared by the dehydration of hemiaminal intermediate, which is produced by a primary amine and an aldehyde or a ketone. Aromatic Schiff base is more stable for stabilizing mechanical properties compared to aliphatic ones (Engel et al., 1985). Zhang et al. (2018) reported a self-healing, pH-responsive, and injectable hydrogel based on aromatic Schiff base using agarose-ethylenediamine and dialdehyde-functionalized polyethylene glycol. The hydrogel exhibited good tissue adhesiveness because of the interaction between the imine bond and tissue protein, which made it a potential candidate for hemostatic material. Figure 2 showed the self-healing process conducted by Zhang et al. (2018).

Besides self-healing, there are many other properties that hydrogels based on imine bonds possess. For example, pH-responsiveness can be achieved because of the nature of Schiff base (Qu et al., 2017). Degradability can be achieved by using chitosan hydrochloride and oxidized dextran to form a dynamic imine bond, because both components could be degraded completely *in vivo* (Jiang X. et al., 2019). Additionally, many of them contain antimicrobial properties. Wang W. et al. (2019) used an ABA triblock copolymer and polyethylenimine to fabricate an antimicrobial self-healing hydrogel. This is achieved because the quaternary amine groups of the copolymer, together with partially protonated amine groups of polyethylenimine, can have electrostatic interaction with the lipid membrane of microorganisms, which is negatively charged, resulting in lysis and cell death. Also, Chen et al. (2019e) studied chitosan-alginate hydrogel and used it for sustained 5-fluorouracil delivery. Targeted therapy was realized by incorporating a magnetic microspheres-loaded anti-cancer drug into the hydrogel scaffold.

#### Boronate-Diol Complexation

Boronate-diol complexation is also referred to as a boronate ester bond (Liu and Hsu, 2018) because it is made from a boronic acid and a diol. The stability of the boronate-diol bond mainly depends on the pH of the solution (Yan et al., 2004). Some researchers (Zhi et al., 2018) combined this kind of bond with bioconjugate chemistry and filamentous viruses to prepare a hydrogel system, which was shown to be stimuli-responsive, injectable, and self-healing. The researchers first conjugated PBA derivative with the M13 virus, and then poly(vinyl alcohol) was used to make cross-linkage with the PBA-M13 via boronate-diol complexation. In addition, the hydrogel exhibited good sugar responsiveness at physiological pH, making it a candidate for release-controlled insulin delivery. Other researchers (Pan et al., 2018) prepared a self-healing and ultra-flexible hydrogel based on hydrogen bonding and boronate-diol ester bond. The hydrogel was prepared with borax, guar gum, and glycerol, forming a glycerol-water-borax net. The dynamic interaction of the net could act as sacrificial bond energy for stretchability and injectability. Due to the stable network of dynamic chemical bonds, the hydrogel could achieve 92.9% healing efficiency of stress and 98.8% healing efficiency of strain when it was put under 25°C for 24 h, and the healing efficiency does not decrease obviously when surface aging happens, including erosion by acid, alkali, or salt. Wu et al. reported a self-healing hydrogel with a wide healing pH range (from 8.5 to 1.5) based on a dual-crosslink network (Wu et al., 2019). One of the crosslinks was based on a rigid nopoldiol-benzoxaborolate bond and the other on a sugar-benzoxaborolate bond. The researchers used several other common diols to form a single network with MAAmBO (the benzoxaborole provider) to compare with nopoldiol, and they were found to be either not dynamic enough or to only have a narrow pH range. Thus, the hydrogel composed of nopoldiol is promising to be used under acidic environment. The dual-crosslink system makes the hydrogel recover very rapidly. Without external forces, two cubes of hydrogel could self-heal in 20 s. Chen et al. (2019f) reported a self-healing hydrogel based on benzoxaborole-diol complexation on nanointerfaces. Interestingly, the “diol” was provided by the galactose residues on the nanosurface of the nanogel, which is temperature-responsive. The naonogel part endows the hydrogel with the same property. This is a promising method to prepare multifunctional hydrogels.

#### Acylhydrazone Bond

Acylhydrazone bond is another reversible chemical bond used in self-healing hydrogels (Yang et al., 2017; Shen et al., 2019; Sun C. et al., 2019). It is formed by combining either acylhydrazine and aldehydes, or acylhydrazine and ketones (Tu et al., 2019). Like the imine bond, an acylhydrazone bond can go through hydrolysis and exchange reactions (Nguyen, 2003). Based on this mechanism, the authors (Yang et al., 2017) prepared a dual responsive hydrogel with very good self-healing efficiency. Carboxyethyl cellulose-graft-dithiodipropionate dihydrazide was mixed with dibenzaldehyde-terminated PEG solutions and 4a-Phe to form the hydrogel, which could potentially be used for three-dimensional (3-D) cell culture scaffolds and drug delivery systems. Without any external intervention, the hydrogels could completely recover themselves within 6 h. In fact, the more 4a-Phe was added in the synthesis of the hydrogel, the better healing efficiency was achieved. This is because the acylhydrazone bonds can exchange in a slightly acidic environment (pH = 4.0–6.0), but they were kinetically locked in neutral conditions (Deng et al., 2012; Wei et al., 2015). 4a-Phe, which is a nucleophilic catalyst, can dramatically increase the exchange reaction. This effort partly addressed the acylhydrazone bond’s problem of only being self-healable in a mildly acidic environment.

#### Metal-Ligand Coordination

**FIGURE 3:**
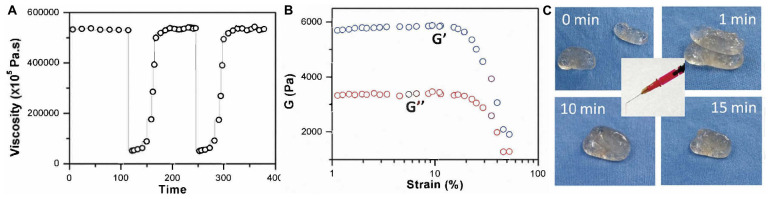
**(A)** Viscosity measurements; **(B)** Strain sweep measurements; **(C)** Self-healing process (reproduced with permission from Chen et al. (2019a). Copyright 2019 Springer Nature).

Metal-ligand coordination is also called chelation (Carlin, 1988). A coordinate bond is a special kind of covalent bond because only the ligand, which is often organic and can also be called a chelating agent, donates electrons instead of one electron from each atom. Silver, iron, calcium, and aluminum ions are common candidates to form the chelation in preparation of self-healing hydrogels. Chen et al. (2019a) studied the coordination between silver ions and S. An injectable, self-healing hydrogel was prepared by simply mixing 4-arm-PEG-SH and silver nitrate (AgNO_3_) aqueous solution. The rheological properties were determined by viscosity measurement. The result was 532,342 ± 2,616 at lower shear rates and 63,056 ± 15,113 at higher shear rates, indicating a high recovery rate of the hydrogel’s structure. Meanwhile, the elastic modulus (G’), which was ∼5700 Pa, and the loss modulus (G”), which was ∼3300 Pa, could both withstand ∼50% strain increase. Also, when cut into two fragments, they could fuse and return to their original state in 15 min at room temperature, indicating a self-healing capacity of the prepared hydrogel. Figures 3A–C show the rheological properties, tensile strength, and self-healing process.

### Physical Non-covalent Interactions

#### Hydrophobic Interaction

Hydrophobic interaction is a typical physical non-covalent interaction. When hydrophobic chains are exposed to water, they tend to aggregate in order for their hydrophobic parts to avoid contact with water as much as possible. In many recent cases, hydrophobic interaction has been used along with other types of cross-linkages to synthesize self-healing hydrogels, such as metal-ligand coordination (Tang et al., 2018), ionic interaction (Sun and Deming, 2019), or even through multiple ones (Deng et al., 2019). Liu P. et al. (2019) studied a thermo-responsive hydrogel made from poly(lactic-co-glycolic acid)-PLGA-PEG-PLGA copolymer, which is widely used in drug delivery (Joo et al., 2009), and was the first to discover the self-healing property of this polymer. The experiment showed that once strain was reduced, the gel quickly recovered almost to its initial state in 10 s.

#### Hydrogen Bond

Hydrogen bond, or H bond, is formed between a hydrogen atom and another atom, which is known as the hydrogen bond acceptor and has a lone pair of electrons. One quality of the H bond is thermo-sensitivity, and thus it has great potential to be used to achieve temperature-responsive self-healing hydrogels. 2-ureido-4[1H]-pyrimidinone (UPy) is extensively used for multiple hydrogen-bonding formation, taking advantage of the quadruple hydrogen bonds. Chen et al. (2019c) prepared a self-healing hydrogel using UPy. Firstly, a hydrogel composed of UPy and negatively charged poly(4-styrenesulfonate) was prepared via one-pot free radical polymerization. After heating and cooling with added polyaniline, the hydrogel was immersed into a solution of acid and FeCl_3_, and through electrostatic interaction the hydrogel was crosslinked with polyaniline. The prepared hydrogel could achieve self-healing within 30 s, and possessed thermoplasticity because of the H bonds. Kuddushi et al. (2019) reported a hydrogel with exciting load-bearing, dye-absorbing, stimuli-responsive, and self-healing properties. This hydrogel was prepared by using a morpholinium-based ester-functionalized surfactant. The self-healing property was determined by observing the cut segments recombining within 12 h in ambient conditions without any external stimuli. Diffusion of methyl orange from doped to undoped gel pieces was also observed during the formation of the self-supporting bridge, which was made up of five gel pieces. Tensile strength comparison between the gels before and after 12-h self-healing demonstrated self-healing efficiency. The self-healed gel could bear 300% stress without interface fracture, and the self-healing efficiency was 78.68%.

#### Host-Guest Interaction

**FIGURE 4:**
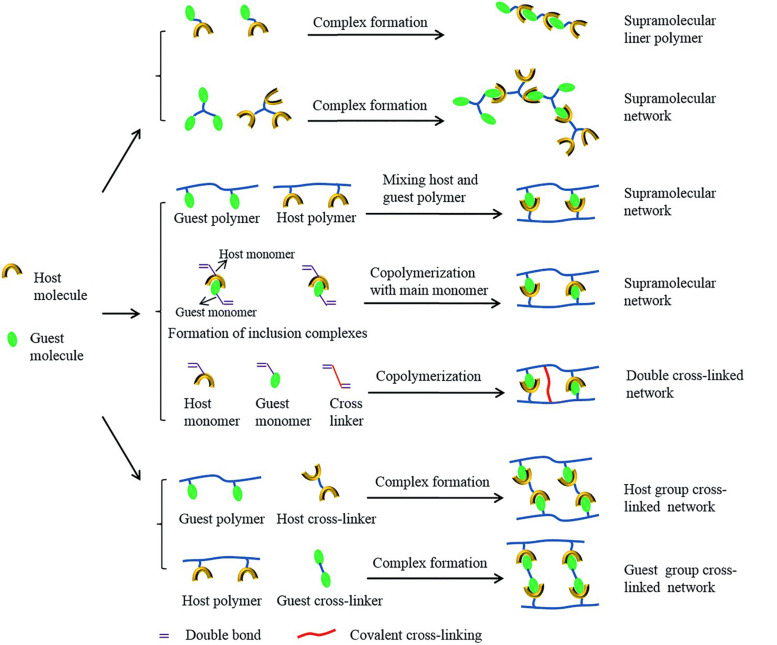
Formations of polymer architectures with host-guest interaction (reproduced with permission from Yang et al. (2015). Copyright 2015 The Royal Society of Chemistry).

Supramolecular host-guest interaction is a non-covalent force between two components of a compound consisting of a host molecule and a guest molecule or ion. The formations of polymer architectures with host-guest interaction are summarized in Figure 4 (Yang et al., 2015). Common molecules used to form host-guest interaction include crown ethers (Xue et al., 2015), calixarenes (Yan et al., 2012), cucurbiturils (Appel et al., 2012), pillararenes (Ogoshi et al., 2016), and cyclodextrins (CDs) (Deng Z. et al., 2018; Li Q. et al., 2018; Liu L. et al., 2019; Wang et al., 2019e; Wu et al., 2008). The authors (Li Q. et al., 2018) used β-cyclodextrin and N-vinylimidazole to achieve hydrogel fibers. The self-healing experiment showed that the cut hydrogel fibers could adhere together within 24 h, and the healed fiber could withstand a reversible stretch. The self-healing efficiency was measured through strain-stress experiments. The originally prepared fibers displayed strength of 0.047 MPa, and the healed ones could achieve 0.045 MPa, indicating an 84% self-healing efficiency. The authors (Wang et al., 2019e) developed a host-guest supramolecule with 3 arms, which were cross-linked with a polymer, and prepared a hydrogel with improved mechanical properties, 3-D printing capacities, and self-healing properties. The supramolecule was prepared via host-guest interaction between modified β-cyclodextrin and modified adamantane. Then, the arms of it went through copolymerization with gelatin methacryloyl to form the hydrogel. Experiments showed that the two pieces of the gel could achieve self-healing after being in close contact for 1 h, and the healed hydrogel would not separate even under a large tensile force. Continuous cyclic deformation was also used to examine the dynamic properties of the hydrogel, showing that when the gel was under a lower shear strain for 140 s after the higher one, the initial modulus value could achieve an almost full recovery. Also, the self-healing efficiency could be controlled when the concentration of the host-guest supramolecule increased, and the efficiency could rise to 80%.

#### Ionic Interaction

Ionic interaction, or ionic bonding, is formed due to the electrostatic attraction between oppositely charged ions, so it is also known as electrostatic interaction (Wang and Heilshorn, 2015). One quality of the hydrogels based on such an interaction is conductivity. Wang Y. et al. (2018) prepared a self-healing, conductive, and metal adhesive DN hydrogel based on ionic interaction and hydrogen bonds by one-pot synthesis. The researchers conjugated graphene oxide with soluble starch and poly(sodium 4-vinyl-benzenesulfonate-co-N-(2-(methacryloyloxy)ethyl)-N,N-dimethylbutan-1-aminium bromide) via γ-radiation. The ionic interaction made the hydrogel not only self-healable but also conductive. When the gel was cut into two parts and brought together, they could achieve ultrafast self-healing without any external stimuli. According to the rheological recovery tests, the storage modulus (G’) of the self-healed hydrogel could nearly be regained within 80 s, indicating good self-healing ability. Also, the ionic conductivity of the healed gel could still achieve more than 80% of its original value after 10 cut-healing cycles.

#### π-π Stacking

π-π stacking happens between aromatic rings. The attraction forces between different electron clouds in an aromatic system form the stacking force (Mcgaughey et al., 1998). Many recent works combined π-π stacking with other covalent bonds or non-covalent interactions to prepare self-healing hydrogels (Liao et al., 2017; Gavel et al., 2018; Liang et al., 2019; Xu H. et al., 2019). Liang et al. (2019) prepared a hydrogel for wound dressing based on the hydrogen bonding and π-π stacking by mixing hyaluronic acid-graft-dopamine with reduced graphene oxide in an H_2_O_2_/HPR (horseradish peroxidase) system. A rheology test was performed to measure the self-healing ability. The continuous step strain test showed that after the first high strain (2000%), the hydrogel network collapsed, and the storage modulus (G’) of the recovered gel decreased largely, while G’ < G” (loss modulus). When at low strain, the G’ partially returned, showing that the hydrogel was partially re-crosslinked. After 4 cycles, nearly the same G’ and G” values, as in the second cycle, could be achieved, indicating the self-healing process. Xu J. et al. (2019) prepared a self-healing hydrogel for strain and pressure sensors based on hydrogen bonding and π-π stacking. The researchers first mixed sodium casein, polydopamine, and acrylamide at 40°C and then used potassium persulfate and N,N,N′,N′-tetramethylethylenediamine as radical initiators and triggered a free-radical polymerization at room temperature. Finally, the samples were molded and placed at 40°C for 5 h to obtain the hydrogel. The synthesized hydrogel could display ultrasensitive resistance responsiveness. Because of the sodium casein, which possesses many amino acid residues with hydrophilic and hydrophobic blocks, the hydrogel was also adhesive. Thus, it is ready to be used for human motion monitoring.

#### Other Physical Non-covalent Interactions

Other physical non-covalent interactions include dipole-dipole association (Sinclair et al., 2018; Wang L. et al., 2019, Wang et al., 2019f), supramolecular donor-acceptor interaction (Li Z. et al., 2018), and so on. Here, we introduce dipole-dipole association as the mechanism of self-healing hydrogels.

**FIGURE 5:**
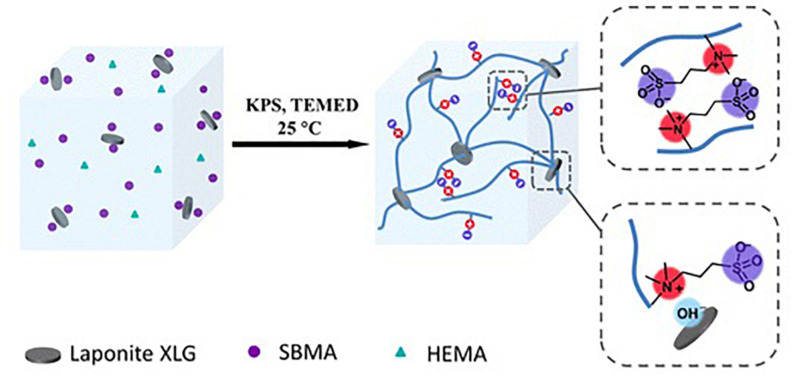
Illustration of a hydrogel based on dipole-dipole association (reproduced with permission from Wang L. et al. (2019). Copyright 2019 American Chemical Society).

The dipole-dipole association can be formed from zwitterionic polymers, molecules that contain a pair of cationic and anionic groups (Sinclair et al., 2018) to remain neutral. The two opposite charges of the molecules make a strong dipolarity, and thus the hydrogel can have good adhesion to many surfaces (Wang L. et al., 2019). The association of polymers can also provide physical cross-linking, and thus mechanical properties can be improved (Talebian et al., 2019). Zwitterions can help ions transport along the depolarized skeleton, making the hydrogel conductive (Wang et al., 2019c). Thus, based on dipole-dipole associations, a conductive, strong, and adhesive hydrogel is able to be prepared. Figure 5 shows a hydrogel based on dipole-dipole association prepared by Wang L. et al. (2019).

### Other Self-Healing Mechanisms

Another mechanism is to take advantage of the properties of components. For example, the structure of some materials changes with temperature. A self-healing hydrogel based on an IPN made up of polyacrylamide (PAAM) and gelatin was developed by Lee et al. (2018). The self-healing behavior was based on the gelatin’s denaturation with temperature changes. The cut pieces of the gel could self-heal to its original form if kept for 1 h at 60°C and cooled down. At high temperatures, free coils will be generated from denatured gelatin and gain mobility according to the second law of thermodynamics (Cuppo et al., 2001; Gornall and Terentjev, 2008). During the cooling process, gelatin froze and the reversible triple helix structure re-networked (Huang et al., 2016), and the new physical entanglement induced the self-healing process.

### Multi-Mechanism Cross-Linking

Recently, many self-healing hydrogels depend not only on a single kind of mechanism, but on two or more different types of mechanisms. The related mechanisms can combine several dynamic chemical covalent bonds; for example, combining imine bonds with acylhydrazone bonds (Wang L. et al., 2018) or with boronate-diol complexation (Balakrishnan et al., 2019). On the other hand, the rest combines different physical non-covalent interactions, such as combining hydrogen bonds with ionic interaction (Long et al., 2018; Jiang X. et al., 2019; Li S. et al., 2019; Lin et al., 2019; Song D. et al., 2019; Yuan et al., 2019; Zhang et al., 2019), with π-π stacking (Liao et al., 2017; Gavel et al., 2018; Liang et al., 2019; Xu J. et al., 2019), and others (Chen et al., 2019b; Qiao et al., 2019), combining ionic interaction with hydrophobic interaction (Chen et al., 2019b), or combining multiple non-covalent interactions (Wang S. et al., 2018; Deng et al., 2019). Additionally, some belong to both categories, such as using both boronate-diol complexation and hydrogen bonds (Ding et al., 2018; Peng et al., 2019; Shao et al., 2019; Wang M. et al., 2019), both imine bond and hydrogen bonds (Liu et al., 2018; Cheng et al., 2019), both host-guest interaction and boronate-diol complexation (Yang et al., 2019), both metal-ligand coordination and hydrophobic interaction (Tang et al., 2018), both dipole-dipole association and boronate-diol complexation (Chen Y. et al., 2018), and multiple ones (Jing et al., 2018; Qu et al., 2018; Pan et al., 2019; Xu H. et al., 2019).

Recently, mussel-inspired chemistry has been extensively studied. The mussel-inspired hydrogels typically consist of polydopamine (PDA), based on which multiple interactions can happen, including hydrogen bonds, π-π stacking, and, mainly, metal-ligand coordination between metals and catechol groups from 3,4-dihydroxyphenyl- L-alanine, an amino acid (Li et al., 2015; Wang W. et al., 2018). Additionally, PDA has abundant functional groups and thus can be easily modified (Fan et al., 2019). Sun Z. et al. (2019) developed a hydrogel based on hydroxypropyl guar gum and dopamine-coated graphene oxide. To prepare the hydrogel, PDA coatings were used to adhere and reduce graphene oxide to obtain nanocomposites. Then hydroxypropyl guar gum and the nanocomposites were mixed in the glycerol/water solvent, while borax solution was gradually dropped into the mixture to form the hydrogel. Because of the multiple interactions, the hydrogel could recover immediately after 1000% strain deformation and also had the ability to detect human motion to large scales from −20 to −30°C.

## Mechanism of Extrinsic Type Self-Healing Hydrogels

Healing agents are stored in reservoirs and embedded in the polymer matrix to achieve self-healing hydrogels, which are called extrinsic type self-healing hydrogels, compared with the intrinsic ones previously mentioned. Microcapsule (Song et al., 2013; Chen et al., 2019d; Liu S. et al., 2019; Rao et al., 2019), microvascular (Toohey et al., 2007), and hollow fibers (Fifo et al., 2014) are widely used reservoirs for storing and releasing healing agents. Among them, microcapsules are especially researched and have the most potential because they are easy to incorporate into the matrix and the microencapsulation technology is already mature (Zhu et al., 2015). The main healing chemistries applied for self-healing materials based on healing agents stored in microcapsules include ring-opening metathesis polymerization (most extensively researched), anionic ring-opening polymerization, cationic polymerization, polycondensation, and free radical polymerization. Based on factors such as external environment, the most suitable chemistry can be chosen (Zhu et al., 2015).

A recent trend in research has been to try to combine extrinsic and intrinsic mechanisms, for example, by modifying the surfaces of the reservoirs. Chen et al. (2019d) reported a self-healing hydrogel based on both hydrogen bonds and a healing agent. To synthesize the hydrogel, STMS was first wrapped by polydopamine (PDA) to achieve functional microcapsules, in which it was then loaded with healing agents, as well as photo-initiators. Finally, poly(methyl-2-ureido-4[1*H*]-pyrimidinone-*co*-butyl acrylate) was used to modify their surface. The experiment showed that the 2 hydrogel pieces could heal together within 4 h at an ambient temperature, and the healed hydrogel could withstand a 200 g tensile load.

## Applications of Self-Healing Hydrogels

Being mostly biocompatible due to their high-water content, hydrogels are always one of the top choices for biomedical uses. Thanks to their ability to recover their structure after damage, self-healing hydrogels are competitive candidates for pharmaceutical, biomedical, and other applications. Self-healing capability in natural tissues is a fascinating property, which can extend life span and enhance reliability and durability (Wang M. et al., 2019). Some self-healing hydrogels also have other properties, such as conductivity, strong adhesion, and stimuli-responsiveness, enabling them to be used in more specific fields. Table 1 summarizes the main categories of the applications of self-healing hydrogels.

**TABLE 1:** Several main categories of the applications of self-healing hydrogels.

Potential application	References
Artificial tissue	Deng Y. et al., 2018; Deng Z. et al., 2018; Ghanian et al., 2018; Lee et al., 2018; Chen et al., 2019b; Guo Z. et al., 2019; Shao et al., 2019; Sun C. et al., 2019
Drug/cell delivery	Qu et al., 2017; Yang et al., 2017; Deng Z. et al., 2018; Khan et al., 2018; Sharma et al., 2018; Wang L. et al., 2018; Wei and Gerecht, 2018; Zhi et al., 2018; Balakrishnan et al., 2019; Chen et al., 2019e; Guo B. et al., 2019; Jiang F. et al., 2019; Li Q. et al., 2019; Liu P. et al., 2019; Qian et al., 2019; Shen et al., 2019; Sun C. et al., 2019; Xu H. et al., 2019
Electrical devices	Liao et al., 2017; Ding et al., 2018; Jing et al., 2018; Li Z. et al., 2018; Li R. et al., 2019; Pan et al., 2018; Shao et al., 2018; Wang S. et al., 2018; Wang L. et al., 2019, Wang M. et al., 2019; Becher et al., 2019; Chen et al., 2019c; Deng et al., 2019; Han et al., 2019; Hu et al., 2019; Lin et al., 2019; Peng et al., 2019; Qiao et al., 2019; Qin et al., 2019; Rao et al., 2019; Shin et al., 2019; Sun C. et al., 2019; Xu J. et al., 2019; Zhang et al., 2019
Glues	Hong et al., 2018; Wang Y. et al., 2018
Wound-healing/tissue regeneration	Nejadnik et al., 2014; Shi et al., 2017; Chen H. et al., 2018; Chen et al., 2019a; Qu et al., 2018; Tian et al., 2018; Zhang et al., 2018; Cheng et al., 2019; Chouhan et al., 2019; Jiang X. et al., 2019; Liang et al., 2019; Wang et al., 2019b
Others	Gavel et al., 2018; Li Z. et al., 2018; Tang et al., 2018; Huang et al., 2019; Kuddushi et al., 2019; Song D. et al., 2019

### Cell/Drug Delivery

The injectability or stimuli-responsive property of many self-healing hydrogels makes them potential candidates for pharmaceutical applications, such as cell or drug delivery.

Being glucose-responsive and injectable at a physiological pH, the hydrogel based on poly(vinyl alcohol), M13 virus, and PBA developed by Zhi et al. (2018) was suitable for controlled insulin delivery. A tumor responsive self-healing prodrug hydrogel was developed by Balakrishnan et al. (2019). This hydrogel had an *in situ* gelling property and is pH- and glucose-responsive, meaning this hydrogel can achieve a triggered release of doxorubicin, an antitumor drug, and thus realize targeted therapy. When the doxorubicin prodrug was doped with miltefosine, a synergistic effect on the inhibition of drug-resistant MDA-MB-231 triple-negative breast cancer (TNBC) cells could be observed.

Cell therapy is becoming a more and more promising treatment. Many injectable and cytocompatible self-healing materials are potential candidates for cell delivery. After exposure through a needle without scaffold protection, the cell viability will be largely reduced (Zhang et al., 2001; Kong et al., 2003). Being able to protect cells, the hydrogel developed by Wei and coworkers was ready for the delivery of ECFCs, a kind of endothelial progenitor cell (EPC) (Wei and Gerecht, 2018).

Smithmyer et al. (2018) developed a boronic acid-based hydrogel, which was suitable for cell culture and complex material construction. The cytocompatibility of the hydrogel made the two encapsulated types of cells able to exhibit good viability over 7 days when cultivated in serum-containing media at 37°C, 5% CO_2_ incubator.

### Glues

Some self-healing hydrogels are adhesive and have other properties, like being stimuli-responsive or conductive. These characteristics mean they could have possible uses in biomedical applications of special glues.

Hong et al. (2018) reported a hydrogel with stimuli-responsive (including glucose-responsive and pH-responsive), remoldable, pressure-sensitive, and adhesive properties. After feeding the hydrogel to mice, increased *in vivo* retention in the intestine region could be observed 24 h after administration. This is due to the mucoadhesive property of the hydrogel, which makes it a potential candidate to be applied as stretchable, self-healing, and multi-responsive biological glues to biomedical platforms.

A hydrogel with conductivity, low cytotoxicity, metal adhesion, and self-healing properties was prepared by Wang Y. et al. (2018). The hydrogel was very adhesive to metal and organic substrates because of the internal adhesive components, which endows the potential application of glues for electronic biomedical products.

### Sensors

Resembling biological soft tissues, conductive hydrogels have the potential to improve wearable health monitoring sensors and electronic skins of soft robots. Because flexible and wearable sensors tend to suffer abrasion and damage easily, the self-healing property can extend the lifespan of them.

Xu J. et al. (2019) designed a strain and pressure sensor with a self-healing hydrogel. This hydrogel, at a pH of around 7.0, had a tough and reusable adhesive behavior. Results also showed that the hydrogel was fatigue resistance and had conductivity. Most importantly, the electric resistance of the gel could increase smoothly and immediately after undergoing increasing tensile strains. The sensor could thus respond to both large-scale and subtle motions, such as saying different words when adhered to the throat, deep breath when adhered to the rib cage, different bending speed of the knuckles when adhered to them, etc. When it comes to pressure sensing, the hydrogels could distinguish standing, jumping, and walking when installed in the volunteer’s shoes, without any large change in electric resistance during repeated motion.

### Wound Repair

The ideal hydrogels for skin repair are those that are able to endure external strain, exhibiting self-healing properties, and preferably with drug-loading capacities for therapeutic purposes (Chen et al., 2019a).

Chitosan (CS) has properties such as being antibacterial, pain-relieving, and hemostatic, and it can be easily modified and cross-linked (Jayakumar et al., 2011; Xu et al., 2015; Lu et al., 2017). Chen H. et al. (2018) prepared an adhesive and antibacterial self-healing hydrogel with CS via a cross-linker. The cross-linker was an oxidized polysaccharide: oxidized konjac glucomannan. Animal experiments showed that the hydrogel could help the wounds of rabbits fully heal 4 days earlier than CS, which was much earlier than the control group. Histological examinations indicated that the gel could accelerate the re-epithelialization of damaged tissues.

Wang et al. (2019b) reported an injectable and antibacterial hydrogel to repair diabetic skin wounds, which was based on polypeptide with stimuli-responsive adipose-derived mesenchymal stem cells exosomes release. According to the animal experiments, the neovascularization and cellular proliferation of treated areas were improved, while tissue formation, re-epithelialization, and collagen remodeling were faster. Meanwhile, Chen et al. (2019a) also prepared an injectable hydrogel for diabetic skin wound repair. This hydrogel was incorporated by desferrioxamine (DFO), making it angiogenic. Moreover, the hydrogel was based on an Ag-S coordination bond, and the silver ions exhibited an antibacterial property. The hydrogel could help the wound heal in 14 days, according to animal experiments.

### Others

In addition to pharmaceutical and biomedical applications (for example, drug delivery and tissue regeneration), some self-healing hydrogels with interesting properties can also be used in other areas, such as batteries (Huang et al., 2019), in environmental protection (Kuddushi et al., 2019; Song D. et al., 2019), in food spoilage monitoring (Tang et al., 2018), and so on. Some applications take advantage of the self-healing property directly, while others are improved due to the self-healing property, for example, lifetime extension.

Huang et al. (2019) reported a self-healing ZIB which could improve the durability of devices and decrease electronic waste. The battery was based on a poly(vinyl alcohol)/zinc trifluoromethanesulfonate hydrogel, which was prepared by a freezing/thawing strategy. Experiments showed that the battery had good consistency. After cutting and self-healing, the electrochemical performance could nearly recover completely, making it possible to be tailored into complicated shapes and patterns, and thus able to be recycled.

Song D. et al. (2019) prepared a self-healing hydrogel with the capability of scavenging heavy metal ions. The hydrogel was based on PA and chitosan (CS). Utilizing the coordination ability of PA, the PA/CS hydrogel showed the ability to capture heavy metal ions, such as Pb^2+^ and Cd^2+^. The porous structure could also facilitate water to penetrate the polymeric network, making the scavenging even better as a result. According to experiments, the scavenging capacity of hydrogel was 1750.66 mg per gram for Pb^2+^ and 1772.4 mg per gram for Cd^2+^, in 12 h.

Tang et al. (2018) prepared a self-healing hydrogel with a color change for BA vapors, which will be generated during food spoilage (Hernández-Jover et al., 1997; Erim, 2013). The hydrogel was based on Ni^2+^ and salicylaldehyde benzoyl hydrazone-terminal PEG (2SBH-PEG). SBH and its derivatives are able to detect BAs by means of hydrogen and charge-charge interaction (Tameem et al., 2010; Basheer et al., 2011). Experiments showed a significant color difference between two hydrogels placed near two pork samples stored for 4 days at 4°C and 25°C individually.

### Summary

Many hydrogels have several properties, and thus have the potential to be used in many fields, such as controlled drug release, tissue engineering, actuators, and light-induced pumps (Wang et al., 2019a). Some hydrogels are still in a theoretical phase, and others have already been tested on animals, most of which have a more specific application, focusing on a certain treatment.

## Conclusion

Self-healing hydrogels are soft materials with robust and recoverable properties. Nowadays, many self-healing mechanisms have been used to achieve intrinsic type self-healing hydrogels, and combining several mechanisms is a growing trend to achieve better physical properties. Another trend is to make modifications to the reservoirs of extrinsic type self-healing hydrogels, to take advantage of both intrinsic and extrinsic self-healing mechanisms. Despite the current problems, such as the self-healing properties of some mechanisms being good only at a certain pH or some self-healing processes happening only at a certain temperature, recent solutions have been in progress. For example, some have tried to solve these problems by adding other components or using them as stimuli-responsive factors. Many self-healing hydrogels with fantastic properties, such as adhesiveness, conductivity, injectability, and so on, increase their potential to be used in multiple areas. Some hydrogels have already been tested in more specific fields and treatments for certain diseases. In the future, hydrogels depending on combined self-healing mechanisms may be further studied, and new kinds of polymers may be used. Although there are remaining concerns to be addressed, the prospect of self-healing hydrogels is promising, and they may be further developed for various applications and specific fields.

## Author Contributions

W-EY conceived the initial idea and the conceptualization. W-EY and YO designed the study and participated in the data extraction and analysis. LF, XG, YQ, MW, and ZZ participated in study design, searched databases, assessed studies, and drafted the manuscript. LF wrote the manuscript. W-EY, YO, XG, and YQ revised the manuscript. All authors contributed to the article and approved the submitted version.

## Conflict of Interest

The authors declare that the research was conducted in the absence of any commercial or financial relationships that could be construed as a potential conflict of interest.

## Abbreviations

3-D: three-dimensional
4a-Phe: 4-amino-DL-phenylalanine
AM: acrylamide
BA: biogenic amine
CIT: cross-linking induced response
CS: chitosan
DA: Diels–Alder
DAA: diacetone acrylamide
DFO: desferrioxamine
DN: double network
ECFCs: endothelial colony-forming cells
EPCs: endothelial progenitor cells
HPR: horseradish peroxidase
IPN: interpenetrating polymer network
LA: lipoic acid
PA: phytic acid
PAAM: polyacrylamide
PBA: phenylboronic acid
PDA: polydopamine
PEG: poly(ethylene glycol)
PLGA: poly(lactic-co-glycolic acid)
RPC: retinal progenitor cell
STMS: stellate mesoporous silica
TNBC: triple negative breast cancer
TPE: tetraphenyl ethylene
UPy: 2-ureido-4[1H]-pyrimidinone
UV: ultraviolet
ZIB: zinc-ion battery.
